# Special issue on “Genomics for future medicine”

**DOI:** 10.1038/s12276-018-0117-y

**Published:** 2018-08-07

**Authors:** Yeun-Jun Chung

**Affiliations:** Precision Medicine Research Center, IRCGP, Department of Microbiology The Catholic University of Korea, College of Medicine, Seoul, Republic of Korea

It has been widely accepted that genome analysis has great potential to explain the causes of diseases and to eventually increase our quality of life. Since the completion of the first human genome sequencing in 2003, our understanding of the human genome, including genetic variations that are responsible for phenotypic diversity between individuals, has been enormously elevated. Based on the precise understanding of our genome, a number of new technologies, such as CRISPR-Cas genome editing, have been developed and adopted for diverse clinical genome studies. In particular, the advent of next generation sequencing (NGS) technology has accelerated the expansion of genome research and clinical translation of personal genome analysis, which enables the practice of precision medicine. Currently, the screening of druggable target mutations serves as routine laboratory tests for cancer patients in many hospitals worldwide. More efficient and cost effective mutation screening using NGS-based gene panel analysis targeting disease-related genes is becoming more popular in clinics. Indeed, the South Korean government has provided conditional insurance for druggable mutation screening with the NGS-based cancer gene panel since 2017. Liquid biopsy has also been translated to the clinic for non-invasive follow-up after cancer treatment.

However, the current success is only the first step to achieving genuine future medicine. For example, more evidence is still needed to support the clinical translation of genetic variations that are associated with human phenotypes or diseases. Although single-cell RNA sequencing (scRNA-seq) technology is becoming a more robust tool for high-resolution analysis of individual cells, technical hurdles, such as low capture efficiency, prevent its direct application in the clinic. Patient-derived xenografts (PDX) are excellent in vivo platforms for mutation analysis-based efficacy testing of candidate drugs. However, at least several months are required to build PDX, and it is unclear whether this model is useful for testing immunotherapeutic trials because it does not recapitulate the human immune system. To overcome these hurdles and move one step toward the next generation of precision medicine, more research and evidence will be required. In this special issue, we present a collection of review articles on cutting edge topics in genomics and genome medicine.

In the first article, Dr. Duhee Bang and colleagues introduce technical challenges in scRNA-seq analyses. Single-cell genome analyses offer superior performance for uncovering novel biological insights compared with traditional bulk cell analyses. These techniques have introduced recent technical innovations in single-cell isolation, library preparation, and computational analysis pipelines supporting single-cell transcriptome data analysis. The relative strengths and weaknesses of various scRNA-seq technologies and potential applications of scRNA-seq methods are also discussed. Dr. Charles Lee and colleagues introduce PDX and humanized mouse models for developing and testing immunotherapeutic strategies. Immunotherapy is a promising method to eliminate tumor cells using an individual’s own immune system. Therefore, selecting appropriate animal models to develop or validate preclinical immunotherapeutic trials is highly important for many cancer research programs. They review the concept, history, and applications of cancer immunotherapy strategies in diverse cancers, as well as the generation, application, and limitations of PDX models. They also introduce the concept of and recent advances in humanized PDX, which represents a ground-breaking research platform for cancer immunology. Dr. Gaia Novarino is one of the leading scientists in the genomics of neurodevelopmental disorders. Her group aims to identify the genes underlying inherited forms of neurodevelopmental disorders, especially autism spectrum disorder and intellectual disability. In this review, they introduce recent advancements in genomic analysis of neurodevelopmental disorders. They also introduce successful examples of translational studies demonstrating how personalized medicine can advance the treatment of neurodevelopmental disorders. The following two papers focus on genetic variations. Understanding the biological impact of genetic variations, including both somatic and germline variations, is crucially important for genomic medicine. Dr. Young Seok Ju and colleagues introduce the patterns and mechanisms of structural variations in human cancers. Together with single nucleotide variants, DNA structural variations are commonly detected genetic events during carcinogenesis. Owing to NGS technology and bioinformatics tools, unbiased catalogs of structural variations are emerging from diverse human cancers, providing new and balanced insight into the biological roles of genetic alterations in tumorigenesis, especially the mechanism and role of genomic rearrangements. Dr. Marcel Dinger and colleagues introduce the significance of noncoding functionality in clinical genomics. Although current clinical genome analyses focus on protein coding genes, the noncoding genome also has potential implications for biology and disease. In this paper, the authors comprehensively review the current challenges and opportunities in uncovering the clinical significance of noncoding genomic information and translating its utility into clinical practice.

We are currently in the era of genomic medicine. Therefore, I believe that the review articles in this special issue are timely and informative for EMM readers. Finally, on behalf of EMM, I would like to express our deep appreciation for the effort and time provided by of all the authors to create this special issue.

